# SOCIAL COHESION AFFECTS THE ASSOCIATION BETWEEN FRAILTY AND DISABILITY IN COMMUNITY-DWELLING OLDER ADULTS

**DOI:** 10.1093/geroni/igz038.2876

**Published:** 2019-11-08

**Authors:** Boqin Xie

**Affiliations:** Fudan University School of Nursing, Shanghai, China, China

## Abstract

The relationships between physical frailty and perceived neighborhood social cohesion (PNSC) and functional disability among community-dwelling older adults are poorly understood. This study aims to (1) examine the associations of frailty and PNSC with disability; and (2) evaluate low PNSC as a risk factor in the association between frailty and disability. A sample of 1645 older adults using multi-stage sampling method in Shanghai were randomly selected in this cross-sectional study. Frailty operationalized as Cardiovascular Health Study criteria (OR=2.4, 95%CI 1.16-4.96 for pre-frailty; OR=7.28, 95%CI 3.37-15.73 for frailty) and PNSC measured as Neighborhood Cohesion Scale (OR=1.81, 95%CI 1.23-2.67) were independently associated with basic and instrumental activities of daily living disability. A significant interaction of frailty and PNSC on disability (F (2, 66)=4.31, P=.014) was found, using a two-way analysis of covariance (ANCOVA). Compared to robust individuals with high PNSC, pre-frailty with high PNSC was not significantly associated with disability while pre-frailty with low PNSC was associated with approximate 4-fold increased prevalence of disability (OR=3.87, 95%CI 1.46-10.24, p=.006). Frailty with high PNSC was associated with higher likelihood of disability (OR=6.47, 95%CI 2.35-17.87) and frail individuals with low PNSC stood out with 10-fold increased prevalence of disability (OR=9.94, 95%CI 3.50-28.26). All analyses were controlled for demographical and clinical covariates. Our results suggest high level of social cohesion serves as a buffer against the impact of physical frailty on functional disability. These findings notably imply to the development of interventions for older frail adults from the neighborhood perspective.

